# Towards the standardization of methods of tissue processing for the isolation of mesenchymal stromal cells for clinical use

**DOI:** 10.1007/s10616-021-00474-3

**Published:** 2021-05-10

**Authors:** Elisabeth García-Muñoz, Joaquim Vives

**Affiliations:** 1 Banc de Sang iTeixits, Edifici Dr. Frederic Duran i Jordà, Passeig Taulat, 116, 08005 Barcelona, Spain; 2 Musculoskeletal Tissue Engineering Group, Vall D’Hebron Research Institute (VHIR), Universitat Autònoma de Barcelona, Passeig de la Vall d’Hebron 129-139, 08035 Barcelona, Spain; 3 Departament de Medicina, Universitat Autònoma de Barcelona, Passeig de la Vall d’Hebron 129-139, 08035 Barcelona, Spain

**Keywords:** Multipotent mesenchymal stromal cells, Bone marrow, Wharton’s jelly, Umbilical cord, Adipose tissue, MSCs isolation protocols

## Abstract

Multipotent mesenchymal stromal cells (MSCs) are currently the most extensively studied type of adult stem cells in advanced stages of development in the field of regenerative medicine. The biological properties of MSCs have generated great hope for their therapeutic use in degenerative and autoimmune conditions that, at present, lack effective treatment options. Over the last decades, MSCs have been typically obtained from adult bone marrow, but the extraction process is highly invasive and the quality and numbers of isolated cells is drastically influenced by patient age, medication and associated comorbidities. Therefore, there is currently an open discussion on the convenience of allogeneic over autologous treatments, despite potential disadvantages such as rejection by the host. This shift to the allogeneic setting entails the need for high production of MSCs to ensure availability of sufficient cell numbers for transplantation, and therefore making the search for alternative tissue sources of highly proliferative MSC cultures with low levels of senescence occurrence, which is one of the greatest current challenges in the scale up of therapeutic cell bioprocessing. Herein we (i) present the main isolation protocols of MSCs from bone marrow, adipose tissue and Wharton’s jelly of the umbilical cord; and (ii) compare their qualities from a bioprocess standpoint, addressing both quality and regulatory aspects, in view of their anticipated clinical use.

## Introduction

**Fig. 1:**
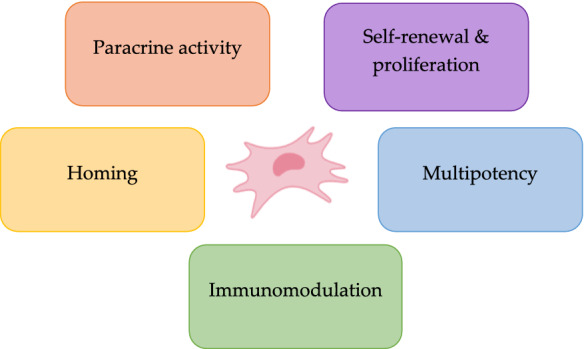
Biological properties of MSC of clinical interest include: (1) trophic function, (2) homing function, (3) immunomodulatory function, (4) multipotency function and (5) self-renewal and proliferation function

Mesenchymal stromal cells (MSCs) are multipotent progenitor cells of mesodermal origin, which are able to self-renew and differentiate into different specialized cell lineages of skeletal tissues, such as: chondrocytes (cartilage cells), osteoblasts (bone cells) and adipocytes (fat cells) (Nombela-Arrieta et al. 2011). They were first isolated from the bone marrow, although they have been also found in a wide variety of adult and neonatal tissues such as adipose tissue, lungs, dental pulp, peripheral blood, placenta and umbilical cord (both from blood and tissue), among others (Sanz et al. 2016; Amable et al. 2014; Kern et al. 2006; Wagner et al. 2005). In fact, recent studies evidenced that MSCs lie adjacent to blood vessels and most likely derive from vascular mural cells known as pericytes, therefore suggesting that MSC could result from any vascularized tissue source (Nombela-Arrieta et al. 2011). The identity of MSC cultures is generally verified by compliance with a set of characteristics known as the “minimal criteria” established by the International Society for Cell & Gene Therapy, including: (i) plastic-adherence of cells that are maintained under standard culture conditions; (ii) expression (≥ 95%) of CD105, CD73 and CD90 markers and lack (≤ 2%) of CD45, CD34, CD14 or CD11b, CD79α o CD19 and HLA-DR markers; (iii) in vitro differentiation potential into osteoblastic, chondrogenic and adipoblastic lineages (Dominici et al. 2006). MSCs have made their mark in the field of cell therapy as promising candidates for the treatment of a wide array of diseases, specifically those of degenerative and immunological origin (Naji et al. 2019). MSCs possess five fundamental biological properties that make them attractive for clinical application: (i) migration into inflamed sites of damaged/diseased tissues when injected intravenously, (ii) promotion of a local immunosuppressive environment, (iii) trophic function through the secretion of bioactive molecules that stimulate tissue healing, (iv) self-renewal and expansion capacity, and (v) differentiation into various specialised cell types (Fig. 1). Importantly, clinical use of MSC has been reported to be safe irrespective of the tissue source, route of administration, dose and condition treated (Thompson et al. 2020; Lalu et al. 2012, 2018). Detailed analyses of the identity, secretome and potency of MSC from different tissues have been widely described in the literature, including back to back comparisons both in vitro and in vivo (Hass et al. 2011; Grégoire et al. 2019). However, even minor changes in the bioprocess design or media formulation can impact on the attributes of MSCs and ultimately on their activity in the patient (Wagner et al. 2007).

The ideal source of MSCs for clinical use must be abundant, easily accessible in order to minimise invasiveness and morbidity, and contain a high number of progenitor cells (Priya et al. 2014). Historically, bone marrow (BM) has been the preferred source of MSCs for clinical use, and this is indeed the best-studied source in the last decades (Friedenstein et al. 1968). However, BM harvesting is a highly invasive procedure as it requires anaesthesia, can cause post-operative pain or discomfort, and exposes the patient to a high risk of nosocomial infections (Naji et al. 2019). Furthermore, it is estimated that MSCs represent only 0.001–0.01% of all nucleated cells present in the bone marrow (Sanz et al. 2016), and their proliferation capacity decline with age of the donor. More recently, adipose tissue (AT) and the Wharton’s jelly (WJ) of the umbilical cord have become alternative sources of MSCs, since they are associated to minimally or even non-invasive procedures (Amable et al. 2014; Kern et al. 2006; Naji et al. 2019). The extraction of MSCs from adipose tissue is performed by liposuction, the most practiced cosmetic procedure nowadays. Despite requiring anaesthesia, this is considered a safe and painless process with a minimal risk of complications for the patient (Haeck et al. 2009). In addition, a greater quantity of starting sample can be obtained, and 1–5% of cells correspond to MSC populations (Naji et al. 2019). On the other hand, the frequency of MSC populations in the umbilical cord is similar or lower than the frequency found in the bone marrow but, in return, they present enhanced expansion potential in vitro due to its foetal origin (Naji et al. 2019). Moreover, its sourcing does not represent any harm to the mother, the foetus or the patient. Medical history of donors has an impact on the qualities of MSC and therefore neonatal sources such as the umbilical cord could result in healthier cells for allogeneic use.

Herein we present and discuss the existing information on different isolation protocols of MSC progenitors derived from BM, AT and WJ.

## Regulatory requirements for tissue sourcing

**Fig. 2:**
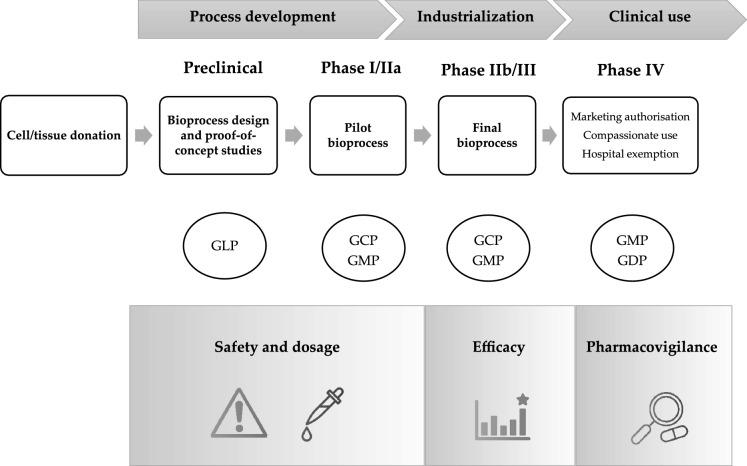
Development programme for cell-based medicinal products. *GLP* good laboratory practices, *GMP* good manufacturing practices, *GCP* good clinical practices, *GDP* good distribution practices

As it happens to any other advanced therapy medicinal product, the development of MSC-based therapies requires strict adherence to regulatory and quality standards (Schneider et al. 2010; Vives et al. 2015). The key steps of such process are summarised schematically in Fig. 2. First, cell and tissue donation must be approved by the local ethics committee and the competent authority (i.e. Organització Catalana de Transplantaments, OCATT, in our territory). Next, in the preclinical R & D phase, a first evaluation of the efficacy, safety and feasibility of the treatment is performed through a series of proof-of-concept studies either in vitro, in vivo or both. Further safety assessment includes the study of the persistence of cells, ectopic differentiation and biodistribution, in compliance with the principles of good laboratory practice (GLP) (Vives et al. 2019). Upon approval by the competent authority (i.e. Agencia Española de Medicamentos y Productos Santiarios, AEMPS, in Spain), clinical-grade production of MSC is performed in clean rooms following procedures compliant with current good manufacturing practice (GMP), and human clinical trials are conducted under good clinical practice (GCP) after approval by the Ethics Committee and conducted in accordance with the Declaration of Helsinki (Gastelurrutia et al. 2021)**.** In phase IV, marketing authorization of the MSC-based product is granted by competent authorities (centralised by the European Medicines Agency, EMA, in Europe). Alternatively, products under investigation can be authorized case-by-case for compassionate uses or, those that are not industrially prepared and are not intended for marketing could be used under the hospital exemption clause for the treatment of specific conditions in approved centres (Roura et al. 2017; Coppens et al. 2020; Cuende et al. 2014; Hills et al. 2020).

## MSC isolation techniques

### Isolation of bone marrow-derived MSCs (BM-MSCs)

BM-MSCs for clinical use are derived from progenitor cells typically isolated from total bone marrow aspirates obtained by puncturing the iliac crest of donors under local anaesthesia. Otherwise total bone marrow can also be obtained from osteotomy of the femoral shafts during orthopaedic surgical procedures (Kern et al. 2006; Wagner et al. 2005; Naji et al. 2019; Hua et al. 2014). The fraction of progenitor cells is further enriched by following either one of the two methods detailed next:

Density gradient centrifugation: a wide variety of protocols describing the isolation of total bone marrow-derived MSCs involve enriching techniques, such as density gradient centrifugation of BM aspirates and later collection of the mononuclear cell fraction, in which MSCs are present together with other types of progenitor cells (Kern et al. 2006; Wagner et al. 2005; Hua et al. 2014; Bieback et al. 2008; Jin et al. 2013). Most methods currently in use employ density gradient media such as Percoll and Ficoll (Bieback et al. 2008). Percoll are silica-based media with a density of 1.130 g/mL that can be adjusted to obtain solutions of different density. Ficoll is a registered trademark solution with a density of 1.077 g/mL ready-to-use for the isolation of mononuclear cells from human peripheral blood (Munteanu et al. 2005). Interestingly, these methods can be automated with the Sepax device (Biosafe, Cytiva) using Ficoll-Paque reagent (Cytiva). To illustrate this application, we followed this methodology in the first step for the production of BM-MSC used in a Phase I/IIa clinical trial for the treatment of gonarthrosis (Codinach et al. 2016; Soler et al. 2016).

Direct plating: although several new devices are available commercial to enrich the mesenchymal cell fraction (Table 2), a very simple protocol for BM-MSC isolation is based on their ability to adhere to the culture plates, by following a direct plating strategy of total bone marrow aspirates to separate plastic adherent MSCs from non-adherent hematopoietic cells. Through this technique, initial cultures present a high hematopoietic contamination (i.e. monocytes), but the MSC fraction is efficiently enriched over subsequent passaging (Bieback et al. 2008). This methodology was successfully used in our laboratory for the production of BM-MSC in a Phase I/IIa clinical trial of spinal fusion in degenerative disk disease (García de Frutos et al. 2020; Vives et al. 2020).

### Isolation of adipose tissue-derived MSCs (ADSC)

ADSC are isolated under anaesthesia by liposuction or lipectomy of the subcutaneous fat tissue located in areas such as the abdomen or the gluteus (Priya et al. 2014; Ghorbani et al. 2014).

Enzymatic digestion: the enzymatic digestion is the conventional technique used for the isolation of ADSC for clinical application. This method involves extensive washes of the lipoaspirate to remove other blood cells, cellular debris and oil, and an enzymatic treatment of the resulting adipose fraction at 37 °C under permanent or intermittent shaking (Amable et al. 2014; Kern et al. 2006; Wagner et al. 2005; Wang et al. 2016). Collagenase is the most widely used enzyme, but its concentration and incubation time can vary according to the protocol (Nombela-Arrieta et al. 2011; Amable et al. 2014; Wagner et al. 2005; Zuk et al. 2001). Once the digestion process is fully completed and the enzyme neutralized, further centrifugation steps are performed to pellet down the stromal vascular fraction (SVF) that contains a heterogeneous mixture of cells including mesenchymal progenitors. Adherent ADSC are separated from all the remaining non-adherent cells by extensive washings after the initial adherence step. As an illustrative example, collagenase treatment was used in the production of ADSC for the treatment of complex cryptoglandular perianal fistula by Garcia-Arranz and collaborators (Garcia-Arranz et al. 2020).

Explant culture: recent studies introduce explant culture as an enzyme-free method for the isolation of ADSC (Priya et al. 2014; Ghorbani et al. 2014). Briefly, the lipoaspirate is extensively washed and allowed to settle down for 5 min to separate the adipose fraction from the aqueous phase. Fat is mechanically fractionated by cutting it into small pieces that are seeded in a culture plate with basal medium at 37 °C with 5% humidified CO_2_. Explants are removed once cells have migrated from the explant tissue to the culture plate. As far as we know, this method is only described in research studies and has not yet been used in any clinical protocol.

## *Isolation of Wharton’s jelly-derived MSCs* (WJ-MSCs)

WJ-MSCs are obtained from the donation of umbilical cords of healthy full-term neonates, after receiving the informed consent of the parents (Salehinejad et al. 2012; Han et al. 2013). Similar to bone marrow and fat, different methods have been developed for the isolation of mesenchymal progenitors. Remarkably, in all cases, ex vivo expanded MSC appear to be equivalent in terms of viability, morphology, rate of proliferation, surface marker expression, levels of cytokine secretion and differentiation capacity (Skiles et al. 2020).

Enzymatic digestion: this technique involves the enzymatic digestion at 37 °C, 5% CO_2_ in a humidified incubator, of umbilical cord fragments (3–5 cm) previously washed after arteries and blood vessel are removed (Amable et al. 2014; Hua et al. 2014; Wang et al. 2016; Salehinejad et al. 2012; Han et al. 2013). Although collagenase is the most widely used enzyme, many laboratories also use trypsin, hyaluronidase or a combination (Hua et al. 2014; Salehinejad et al. 2012). The digestion process conditions may vary according to the protocol, mostly subjected to the enzyme type and concentration employed. Anyhow, once the enzymatic digestion is finished, WJ-MSCs are isolated by adherence, after culturing the resulting pellet. To our knowledge, this technique has only been described in research studies and has not yet been reported in any clinical protocol. However, the MERLIN (MEsenchymal stem cells to Reduce Liver INflammationlarger fragments of the umbilical cord tissue (including WJ) trial reported the use of the enzymatic methods for the preparation of the Master Cell Bank of MSC from larger fragments of umbilical tissue, therefore potentially including the Wharton’s Jelly (ClinicalTrials.gov Id. NCT02997878).

Explant culture: through this method, WJ-MSCs are isolated without the need of any enzymatic treatment. In short, umbilical cord fragments previously washed and with the arteries and blood vessel removed, are mechanically fractionated by cutting them into smaller fragments (2–3 mm) that are directly seeded in a culture plate with basal medium (Wang et al. 2016; Salehinejad et al. 2012). Explants are maintained in a humidified atmosphere with 5% CO_2_ at 37 °C, until cells migrate from the tissue to the culture plate. After that, explants are removed and cultures preserved until they reach 90% confluence (Hua et al. 2014; Salehinejad et al. 2012; Han et al. 2013; Ishige et al. 2009). Specifically, we have investigated the safety and efficacy of WJ-MSC resulting from the use of this methodology in clinical trials for the treatment of inflammatory conditions including chronic spinal cord injury (ClinicalTrials.gov Id. NCT03003364), severe respiratory distress due to SARS-CoV-2 infection (ClinicalTrials.gov Id. NCT04390139) and myocardial infarction in the PERISCOPE (the PERIcardial matrix with mesenchymal Stem Cells fOr the treatment of PatiEnts with infarcted myocardial tissue) trial (ClinicalTrials.gov Id. NCT03798353) (Albu et al. 2021; Prat-Vidal et al. 2020).

## Attributes of adult and neonatal MSCs

Several studies have shown that MSCs derived from bone marrow, adipose tissue and Wharton’s jelly share a very similar immunoprofile that complies with the ISCT’s minimal criteria: they are positive for CD105, CD73 and CD90 and do not present CD45, CD34, CD14 or CD11b, CD79α o CD19 and HLA-DR markers (Amable et al. 2014; Kern et al. 2006; Hua et al. 2014; Jin et al. 2013; Wang et al. 2016; Lee et al. 2004; Heo et al. 2016). All of them also possess multilineage differentiation capacity (Amable et al. 2014; Kern et al. 2006; Wagner et al. 2005; Jin et al. 2013; Wang et al. 2016), although WJ-MSCs appear to take longer times to differentiate in vitro towards osteogenic and adipogenic lineages compared to adult BM-MSCs and ADSC (Amable et al. 2014). However, we observed that factors secreted by BM-MSCs during osteogenic differentiation greatly accelerate osteogenic induction of WJ-MSCs, suggesting that the intra-osseous environment could be sufficient for this population to guarantee a successful bone regeneration, with a similar therapeutic efficacy to that of BM-MSCs (Cabrera-Pérez et al. 2019). Although WJ-MSCs may initially take longer times to reach 80–90% confluence (Hua et al. 2014; Salehinejad et al. 2012; Han et al. 2013; Nagamura-Inoue et al. 2014; Cheng et al. 2011), they are typically cultured for more passages before presenting signs of senescence (Table 1), with higher population doubling levels in comparison to adult BM-MSCs and ADSC (Amable et al. 2014; Hua et al. 2014; Cheng et al. 2011). Indeed, tissue age seems to be a key factor in the proliferative and expansion capacity of the isolated MSCs, being greater the more primitive the tissue is (Stolzing et al. 2008; Jones et al. 2010). This fact was further corroborated by Wang and collaborators (2016), who showed that MSCs derived from foetal bone marrow, a tissue even more primitive than the umbilical cord, present higher rates of proliferation and expansion than WJ-MSCs.

**Table 1:** BM-MSCs, ADSC and WJ-MSCs isolation and expansion characteristics

MSC source	Average time to 80–90% confluency	No. passages	Max No. passage
BM	7 days	P8–P13 (adult) P22–P24 (foetal)	P13 (adult)
AT	10–14 days (Exp)	P8–P13	P20
	3–7 days (Enz)		
WJ	2–4 weeks (Exp)	P17–P18	P25
	12–14 days (Enz)		

WJ-MSCs and foetal BM-MSCs present the highest proliferation capacity. Enzymatic digestion allows faster ADSC and WJ-MSCs isolation compared to the explant culture technique

*BM* bone marrow, *AT* adipose tissue, *WJ* Wharton’s jelly, Exp xplant culture method, *Enz* enzymatic digestion method, *P* passage

## Comparison of isolation methods: explant culture vs. enzymatic digestion

There is still no consensus that determines the most cost-effective protocol for the isolation of MSCs populations. Explant culture prolongs primary culture time, but it is an inexpensive process requiring very little manipulation and resulting in homogeneous cell populations, since only those that can migrate from the tissue to the plastic will proliferate (Priya et al. 2014; Hua et al. 2014; Ghorbani et al. 2014; Salehinejad et al. 2012; Han et al. 2013). On the other hand, enzymatic digestion allows rapid cell isolation, but enzymes increase the price of the process and generate a very high proteolytic stress that may affect cell membranes and consequently, their adhesion capacity and viability (Hua et al. 2014; Salehinejad et al. 2012). Moreover, this technique requires multiple steps leading to a higher risk of biological contamination. Due to the intense degradation of the tissue, cultures from enzymatic digestions are very heterogeneous at initial passages with increased hematopoietic contamination (as a higher percentage of CD34 and HLA-DR positive cells are detected) (Priya et al. 2014; Salehinejad et al. 2012), are morphologically affected and have lower proliferation rates (Hua et al. 2014; Salehinejad et al. 2012). Therefore, from a clinical and regulatory point of view, explant culture would present several advantages comparison to enzymatic digestions (Table 2). However, more studies are needed involving different enzyme types, concentrations and incubation times, since enzymatic digestions not affecting cell adhesion and viability could lead to highly efficient cultures (Priya et al. 2014; Han et al. 2013), which is a key point for large-scale MSCs isolation and expansion. Having said this, the addition of enzymes (mostly from xenogeneic origin) may pose safety risks that must to be assessed thoroughly before submission of the Investigational Medicinal product Dossier (IMPD) to the regulatory authority (Vives et al. 2021).

**Table 2:** Comparison of MSC isolation methods, which can benefit from MSC enrichment techniques

MSC isolation technique	Advantages	Disadvantages
Enzymatic digestion	Rapid MSC isolation	Increased price
		High proteolytic stress on cells
		Increased hematopoietic contamination
		Several processing steps: high risk of biological contaminations
Explant culture	High proliferation rate	Time-consuming
	Cheaper process	
	Do not cause cell membranes damage	
	Homogenous populations	
	Little manipulation: low risk of biological contaminations	
Enrichment techniques	Enrichment of the MSC fraction	Additional step at the start of the scale up production workflow
	Fast process	Additional cell manipulation: increased risk of contaminations and addition of impurities
	Automated (in some cases)	Expensive

## Protocol standardization and optimization needs

Leaving aside differences in MSCs properties according to the tissue source, there are several other factors that may impact on the variability of their ultimate functional characteristics such as the isolation method and MSC enrichment techniques, formulations of the expansion culture medium (considering the use of animal derived supplements or xeno-free/serum-free chemically defined media), environmental conditions and scale up strategy (García-Fernández et al. 2020; Galipeau 2013). For this reason, specifications of final product need to be clearly established in terms of cellular identity, purity and potency according to the expected mechanism of action (Vives et al. 2019). There is currently a lack of global harmonisation in compliance with quality standards and regulatory guidelines for the establishment of GMP-compliant manufacturing protocols to ensure scalable, robust and reproducible production of MSCs that satisfies safety, quality and quantity demands for its therapeutic application therefore ensuring cross-comparison of data resulting from clinical trials using MSC-based products prepared in different laboratories (Naji et al. 2019; Bieback et al. 2008; García-Fernández et al. 2020). Our contribution to ensure consistency of the qualities of MSC in clinical trials is the development and validation of a robust bioprocess design for the scale up of clinical grade WJ-MSC by following a two-tiered cell banking strategy (Master Cell Bank and Working Cell Bank) in compliance with GMP (Grau-Vorster et al. 2019; Oliver-Vila et al. 2016). This methodology has resulted in MSC-based products in conformity with the same specifications that were used in clinical trials and compassionate cases (Albu et al 2021; Prat-Vidal et al. 2020). Moreover, risks such as cell aging, and therefore preservation of MSC’s attributes, were mitigated by ensuring that cumulative population doublings were always under 40, which is considered the safety limit in culture.

## Conclusions

In summary, all three types of MSC cultures deriving from bone marrow, adipose tissue and Wharton's jelly meet the minimum requirements established by the ISCT, that is: they are spindle-shaped adherent cells, they accomplish the established immunogenic profile, and they have the ability to differentiate into osteoblasts, adipocytes, and chondrocytes. However, choosing the best source of MSCs by directly comparing the three population characteristics remains a very difficult task, since a standardized isolation protocol has not yet been defined, and the initial source may not be the only factor that influences the basic properties of resulting MSCs. Consequently, additional comparative studies involving different isolation conditions are needed to promote the development of global standards and manufacturing guidelines. Despite of this, the Wharton's jelly from the umbilical cord hold high expectations and appears to be close to an ideal source for the development of allogeneic cell therapies, as its sourcing does not pose any risk to the mother nor the neonate, and gives rise to more primitive and hypoimmunogenic MSC populations with high proliferation capacity and low senescence rates. Nevertheless, Wharton’s jelly remains the most time-consuming source of primary culture MSCs isolation. The establishment of cost-efficient protocols that allow large-scale expansion of WJ-MSCs without altering their biological properties, is one of the greatest challenges to ensure the progression of this cell type towards cell therapies.
